# In Silico High-Performance Liquid Chromatography Method Development via Machine Learning

**DOI:** 10.1021/acs.analchem.4c03466

**Published:** 2025-03-28

**Authors:** Alberto Marchetto, Monica Tirapelle, Luca Mazzei, Eva Sorensen, Maximilian O. Besenhard

**Affiliations:** † Department of Chemical Engineering, University College London, Torrington Place, London WC1E 7JE, U.K.; ‡ Department of Management, Economics and Industrial Engineering, Politecnico di Milano, Via Raffaele Lambruschini 4/B, Milano 20156, Italy

## Abstract

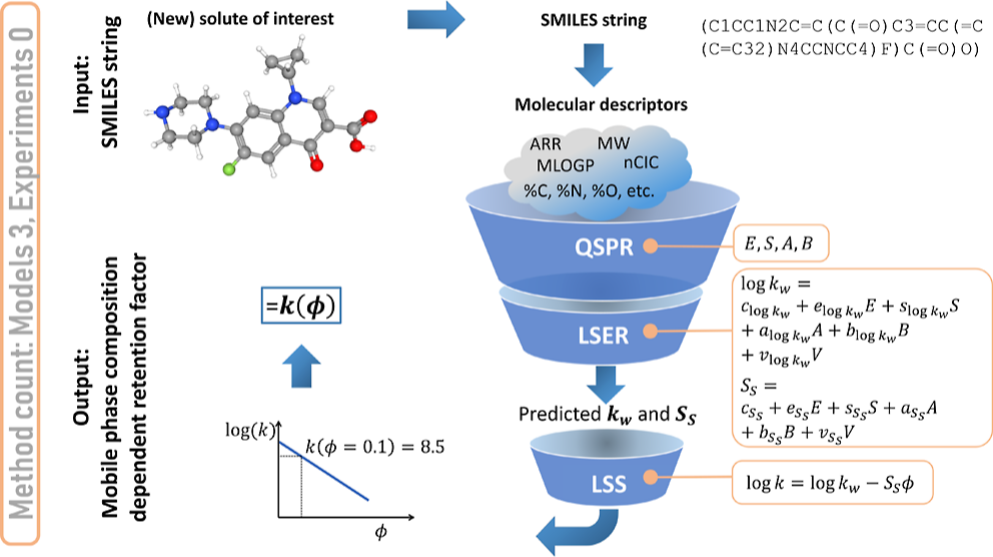

High-performance liquid chromatography (HPLC) remains the gold standard for analyzing and purifying molecular components in solutions. However, developing HPLC methods is material- and time-consuming, so computer-aided shortcuts are highly desirable. In line with the digitalization of process development and the growth of HPLC databases, we propose a data-driven methodology to predict molecule retention factors as a function of mobile phase composition without the need for any new experiments, solely relying on molecular descriptors (MDs) obtained via simplified molecular input line entry system (SMILES) string representations of molecules. This new approach combines: (a) quantitative structure–property relationships (QSPR) using MDs to predict solute-dependent parameters in (b) linear solvation energy relationships (LSER) and (c) linear solvent strength (LSS) theory. We demonstrate the potential of this computational methodology using experimental data for retention factors of small molecules made available by the research community for which the MDs were obtained via SMILES string representations determined by the structural formulas of the molecules. This method can be adopted directly to predict elution times of molecular components; however, in combination with first-principle-based mechanistic transport models, the method can also be employed to optimize HPLC methods in-silico. Both options can reduce the experimental load and accelerate HPLC method development significantly, lowering the time and cost of the drug manufacturing cycle and reducing the time to market. Given the growing number and quality of HPLC databases, the predictive power of this methodology will only increase in the coming years.

## Introduction

High-performance liquid chromatography (HPLC), introduced in the 1960–70s,^1,2^ remains essential in both academia and industry for analyzing and separating molecular components in solutions. It is widely used for applications ranging from biological sample analysis to product purification in industrial processes.^3^ Its high accuracy and versatility make it crucial for chemical and pharmaceutical research and manufacturing. The technique involves a mobile phase (e.g., a solvent mixture setting the polarity) carrying the sample liquid comprising the molecules of interest (i.e., the solutes) through a stationary phase, typically a column packed with small porous particles. The different affinities of the solutes with the stationary and mobile phases (e.g., due to the differences in polarity) determine their retention times in the column, *t*_R_. Stronger interactions with the stationary phase cause solutes to elute (i.e., leave the column) later; that is, the solutes are retained in the column for longer. Unretained solutes, which do not interact with the stationary phase, all elute at the HPLC system-specific dead time, *t*_0_ < *t*_R_. The exit of the column is connected to a detector that can (e.g., along with a calibration curve) quantify the eluting solutes for sample analysis. For purification, the solutes are collected separately. Therefore, achieving well-resolved, time-displaced solute elutions is crucial for effective separation and analysis, making it the primary objective of HPLC method development, particularly in reversed-phase liquid chromatography (RPLC), which is the focus of this work.

The interplay of fluid dynamics, transport phenomena, and adsorption thermodynamics (which affects the solute affinities with the stationary and mobile phases) is complex.^4^ This makes it challenging to develop a suitable HPLC method, i.e., to identify the right HPLC settings, such as stationary phase material, temperature, pH, sample volume, flow rate, and especially mobile phase composition. This is particularly true for samples containing either several solutes or solutes with high chemical similarity. Owing to this inherent complexity, HPLC methods are commonly developed via trial-and-error experimental campaigns, with empirical starting conditions and one-variable-at-a-time strategies often driven by experience. For complex samples or new solutes, these approaches are at high risk of failing, not least because the number of experiments is often limited by the small sample quantities available at early development stages.

## Computational HPLC Method Development

Different computational approaches have been considered for HPLC method development for decades to minimize costly and time-consuming experiments and to gain insight into the separation mechanisms. As the accuracy of these models and computational power continue to improve, simulations have become more integrated into Quality by Design concepts.^5^ Moreover, as machine learning and artificial intelligence become more powerful and integrated into the daily workflow, in-silico HPLC approaches will only become more important.^6,7^

Ideally, HPLC methods can be developed (and directly validated) using digital HPLC twins equipped with models that account for all HPLC settings considered for method development, i.e., models predicting how each of these settings affects the elution behavior.^8^ Models of this kind include, but are not limited to, the equilibrium dispersive model, the lumped kinetic model, and the general rate model.^3,9,10^ These HPLC transport models are derived from first-principles but are usually unclosed. To be solvable, they must be coupled with solute-specific adsorption isotherms or mass transfer rates (which are generally unknown). Adsorption isotherms describe the ratio between the concentration of a solute in the mobile phase and that of the solute adsorbed on the surface of the stationary phase, at equilibrium conditions.^11^ This ratio is not constant but depends on many variables, such as temperature, pH, mobile phase composition, and (in general) solute concentration in the mobile phase. Several adsorption models are available, most of them being semiempirical in nature and featuring parameters that cannot be predicted a priori. Commonly used are the (single, bi- or tri-) Langmuir type and competitive adsorption isotherm models.^12−14^ At a low solute concentration, these reduce to the Henry’s adsorption isotherm, where the solute concentrations in the stationary and mobile phases are linearly proportional. Adsorption isotherm models accounting for temperature and/or pH have been reported.^15−18^ However, these models are rather complex, since they feature many unknown parameters and thus are not commonly used.

The inherently complex relation between solute adsorption and temperature, pH, and type of stationary-phase material is the main reason why these HPLC settings are usually preferred to be kept constant when HPLC methods are developed. Instead, HPLC settings related to the mobile phase composition are often changed first due to the strong impact on the elution behavior and the ease of doing so. A simple way to describe how the mobile phase composition alters the solute adsorption (hence, adsorption isotherms) and in turn the retention times, is offered by the linear solvent strength (LSS) theory.^4,19^ This theory is commonly expressed as
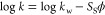
1where *k* ≡ (*t*_R_ – *t*_0_)/*t*_0_ is the solute retention factor, ϕ ranges from 0 to 1 and is the volume fraction of organic modifier (i.e., the least polar solvent component in the case of the commonly used RPLC) in the mobile phase, *k*_w_ is the extrapolated solute retention factor in the lower limit of ϕ → 0 (i.e., in pure water, if water is the polar solvent component in the mobile phase), and *S*_*S*_ is the solvent strength parameter. Owing to its simplicity, the LSS theory is widely adopted, particularly for small molecules but also for peptides and proteins,^20^ to account for the effect of mobile phase composition on solute retention. The LSS theory is, however, known to be less accurate at high volume fractions of organic modifier, which is why nonlinear adaptations have been proposed.^21,22^ It is important to note that the LSS theory does not account for significant changes in pH, as its parameters are specific to a single pH value.

Deriving solute adsorption isotherms solely from first-principles is not possible (yet). Hence, experiments remain essential to identify suitable adsorption models and to calibrate their parameters (e.g., *k*_w_ and *S*_S_, or additional parameters for nonlinear adsorption isotherms and/or solvent strength models). Once the parameters have been estimated, mathematical models are a powerful tool for developing HPLC methods, but the experimental effort required for parameter estimation can be considerable.

## Data-Driven HPLC Models

Because of this effort, data-driven models which predict the solute retention directly, i.e., without needing calibration experiments, are widely recognized. Their accuracy continues to improve in the modern era of high-throughput analysis and machine learning.^23−26^ Most data-driven models relate descriptor variables representative of the molecular attributes of the solutes (inputs) to their retention behavior, for instance *k*, *t*_R_, or retention time indices^27^ (outputs). These models are referred to as quantitative structure-retention relationships (QSRR).^28^ As opposed to QSRRs, QSPRs are models with other physicochemical properties as outputs. Commonly used descriptor variables are convolutional filters or selectors applied to molecular structure representations, molecular fingerprints, or molecular descriptors (MDs).^29−31^ MDs are the transformations of “chemical information encoded within a symbolic representation of a molecule into a useful number”,^32^ and more than 5000 such transformations can be calculated from a molecular structure.^33^ MDs can be determined before a molecule is synthesized once its molecular structure is known. Commonly used QSRR and QSPR methods^28^ include multiple linear regression,^30,34,35^ projection to latent structures (or partial least-squares) regression,^36,37^ decision trees,^38^ random forests,^39^ support vector regression,^40−42^ gradient boosting,^25,43^ Gaussian process regression,^44^ and artificial neural networks/deep learning regression.^23,25,31,45,46^

An approach that is not purely data-driven (i.e., where the equations of the model are at least partly based on physical principles) allows for the prediction of retention behavior based on LSERs, also known as Abraham solvation parameter models.^47,48^ For chromatography, LSERs relate physicochemical properties of solutes as well as HPLC system (i.e., stationary and mobile phase) properties to retention behavior through linear models.^49^ LSERs are a subclass of linear free-energy relationships^50^ and are, technically, a form of QSPRs aiming to predict any free-energy-related property through linear contributions of different interaction abilities affecting the solvation energy of the solutes. For HPLC systems, the free-energy-related property is often the retention factor predicted as
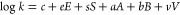
2

The uppercase letters *E*, *S*, *A*, *B,* and *V* denote the LSER solute parameters describing the solute properties, where *E* (excess molar refraction) is a measure of solute refractivity, *S* (dipolarity/polarizability) is a measure of solute dipolarity and polarizability, i.e., the tendency of a solute to form dipole–dipole and dipole–induced dipole interactions, *A* (hydrogen bond acidity) and *B* (hydrogen bond basicity) quantify the tendency of the solute to participate in hydrogen bonds as acid and base, respectively, and *V* (McGowan’s molecular volume) is a measure of the solute molecular volume. The lowercase letters *c*, *e*, *s*, *a*, *b,* and *v* denote the LSER coefficients or chromatographic system parameters. These are independent of the solute and account for the specific interactions between the mobile and stationary phases. These LSER system parameters are commonly determined via parameter estimation from retention experiments (where usually *t*_R_ is measured) using solutes with known LSER solute parameters and a mobile phase with a fixed volume fraction of the organic modifier. This is because the system parameters depend on the type of organic modifier used and on its volume fraction. Hence, they are (unknown) functions of ϕ, and a change in organic modifier will require new LSER coefficients. This means that LSERs in the form of eq 2 are not suitable for the HPLC method development.

Other commonly used computational tools, such as the hydrophobic subtraction model—particularly employed to select stationary phases that maximize selectivity for solutes across various mobile and stationary phase combinations (and analogous approaches)^51−53^—are mathematically similar and hence share comparable limitations when applied to method development. On that note, the concept of combining these models with submodels is very promising, as demonstrated by the integration of the hydrophobic subtraction model and LSS theory with QSRR to predict solute-specific coefficients.^54−56^ Although the potential of these data-driven approaches is well established, they may lack predictive power for new solutes because models are commonly trained on relatively small in-house databases. In addition, databases are commonly based on single chromatographic systems (i.e., same stationary phase, same organic modifier, and same concentration or concentration profile), which makes the models unusable if another system is considered. To map retention times between different chromatographic systems, transfer functions have recently been proposed. An example is the PredRet database, which comprises of retention times for various mobile and stationary phases and HPLC settings.^57^ While merging chromatographic data sets can significantly enhance data-driven models, the accuracy of transfer functions may vary. Nevertheless, this approach is crucial and represents a promising path forward for integrating data obtained from different systems. Although merging chromatographic data sets would empower data-based models, the accuracy of transfer functions is still insufficient. Additionally, well-structured chromatographic databases are growing in size and number due to the significant advances in high-throughput chromatography, not least due to the incentive to better utilize machine learning. Noteworthy is the METLIN small molecule data set, which lists molecular structures and retention times of more than 80,000 solutes, allowing for deep learning-based retention time prediction.^23^ Empowered by such large data sets, data-driven models can predict retention times remarkably well solely based on molecular structure representations.^23,31,42^

Still, even the most comprehensive data-driven models are rarely useful for HPLC method development despite the success of these black (or gray, as they may be difficult to interpret rather than entirely uninterpretable) box models to accurately predict the elution behavior for new solutes. This is because such models are usually trained for only one chromatographic system and for specific HPLC settings. Hence, they fail to predict solute elution behavior if any of the HPLC settings change, including the volume fraction of organic modifier, which is a key variable for optimizing the separation. This is a significant limitation because it precludes the use of these models for method development, i.e., to computationally identify the right HPLC settings. In order to optimize HPLC methods in-silico, models that can predict the elution behavior for changing HPLC settings are needed. It should be noted that this can be achieved with data-driven models trained with solute- and HPLC setting-specific information, i.e., HPLC settings become a model input to allow predictions (e.g., of retention times) for new settings.^46,58^ Such merged models are more flexible but remain impractical as the number of experiments needed for model training can be expected to increase proportionally to the power of the number of HPLC settings considered.

## New Methodology and Article Structure

In this work, we address the shortcoming of data-driven models not being able to predict elution times for varying HPLC settings (without the need for extensive experimentation for parameter estimation) and to relate molecular properties to mobile phase composition-dependent retention behavior. In particular, the approach proposed here provides a promising tool to develop HPLC methods in-silico with optimal mobile phase compositions based solely on the SMILES string representation of solutes.^59^ First, we describe the concept of the data-driven methodology combining multiple data-based strategies. We also outline the details of the QSPR, LSER, and LSS models used. Then, we demonstrate how the methodology was applied and tested and discuss the contribution of each model on the overall performance. And finally, we conclude by commenting on the potential of the methodology and addressing future perspectives. Additional details of data selection and curation, as well as rationales for input variable reduction, are provided in the Supporting Information (S.1–2).

## Data-Driven Methodology for Predicting Solvent Composition Dependent Retention Factors

**Figure 1:**
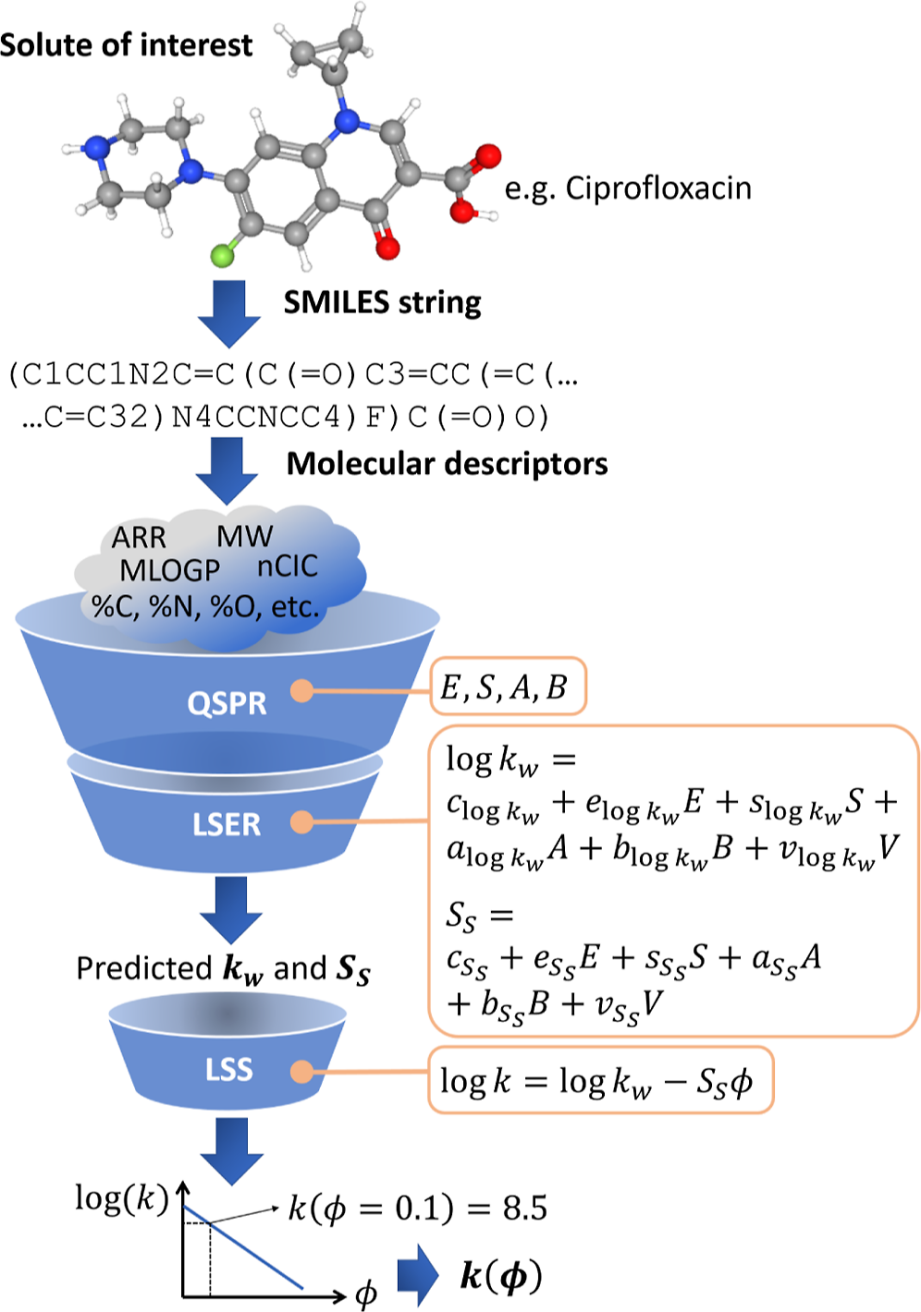
Work flow of data-driven methodology predicting the linear solvent strength model parameters *S*_S_ and *k*_w_ via QSRR and LSER starting from MDs. In turn, these are obtained from the molecular structure provided as SMILES string. The molecular representation of ciprofloxacin is taken from PubChem.

The methodology proposed in this work is listed in Figure 1. The methodology combines (a) QSPR models using MDs to predict (b) LSER solute parameters and (c) LSS theory to link molecular retention behavior with varying mobile phase compositions. The methodology first determines the MDs of the solutes considered from their molecular structures, here obtained from solute molecular SMILES strings.^33^ The MDs are the inputs to four different QSPR models, each predicting one of the following four LSER solute parameters (and not directly retention factors or times): *E*, *S*, *A,* and *B*. The fifth solute parameter present in eq 2, *V*, can be determined directly from the molecular structure.^60,61^ Two LSER models equipped with these solute parameters and the HPLC system parameters (here not limited to a single volume fraction of organic modifier) are then used to determine the specific LSS parameters of each solute, *k*_w_ and *S*_S_ (see eq 1).

The following section outlines the development of the QSPR models, along with the selection of the MDs, the development of the LSER models, and the integration of these models with the LSS theory. This data-driven methodology facilitates the prediction of solute retention factors, considering changes in the mobile phase composition while requiring only knowledge of solute SMILES string representations. This enables in silico optimization of the mobile phase composition for isocratic HPLC methods (ϕ is constant) or, in combination with an HPLC transport model, of the initial and final mobile phase compositions and of its dynamic change for gradient HPLC methods (ϕ varies with time).

### LSER Solute Parameter Prediction via QSPRs

The LSER solute parameters *E*, *S*, *A,* and *B* are usually determined experimentally, which is time-consuming and requires multiple analytical techniques and sufficient material to perform the analyses. A data-driven alternative to obtain these parameters is to use QSPR models with MDs as inputs and rely on existing LSER solute parameter databases for model training. This is feasible thanks to the availability of large databases of experimentally determined LSER solute parameters. Examples are the UFZ-LSER database from the Helmholtz Centre for Environmental Research, which provides LSER solute parameters for more than 7000 small molecules collected from different sources;^62^ the SoluteDB, which contains between 7000 and more than 8000 entries for each LSER solute parameter;^63^ and the Wayne State University experimental descriptor data set, which lists LSER solute parameters for several hundred solutes collected in a single laboratory via standardized procedures, thus minimizing experimental variations.^64^ For this work, we used the so-called Abraham Absolv data set (taken from the UFZ-LSER database) which, at the time of this work, comprised LSER solute parameters for 7881 small molecules. This data set is the result of Abraham’s work in the field of LSER and is used here to build the QSPRs for LSER solute parameter prediction. As detailed in the Supporting Information (Section S.1), this data set was reduced to 6437 solutes by (a) removing molecules with missing values of at least one of the four LSER solute parameters considered, (b) removing molecules with unknown SMILES strings (for which it was not possible to associate any MD), (c) removing duplicate molecules, and (d) narrowing the molecular weight range, i.e., considering only solutes within a molecular weight range of 80–400 g/mol. Additionally, 36 molecules used to demonstrate the principle of our methodology (see Section: Development of LSER) were excluded to guarantee complete independence between methodology development and testing. Hence, 6401 solutes were used for QSPR development.

### Initial MD Selection for QSPR Development

MDs were obtained from the solute SMILES strings via the chemoinformatics software alvaDesc.^33^ Selected MD classes comprised constitutional indices, molecular properties, topological indices, ring descriptors, connectivity indices, 2D autocorrelation descriptors, and Getaway descriptors, yielding a total of 804 MDs. Three-dimensional (3D) MD classes were not selected because SMILES strings contain no detailed information on 3D molecular structures. The selected classes were chosen as they provide information on the structure and physicochemical properties of the solutes. The 804 MDs computed with alvaDesc were reduced to 612 by withdrawing (nearly) constant MDs (see Supporting Information, S.1, for further details) and one MD containing mostly missing values (following a manual inspection).

### QSPR Development

To best predict *E*, *S*, *A,* and *B*, four QSPRs were developed (one for each LSER solute parameter), using least-squares regression with weight decay regularization, i.e., ridge regression.^65,66^ Ridge regression is based on a linear relationship between input and output variables, which in principle can limit QSPR prediction capabilities.^67^ However, nonlinear models based on artificial neural networks were also tested, but despite their higher complexity, they did not improve the predictive performance significantly compared to ridge regression (results not shown for sake of brevity). Additionally, the relatively simple ridge regression method reduces the risk of overfitting (also thanks to weight decay regularization, as explained below). The loss function *J* that ridge regression minimizes can be expressed as
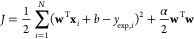
3where *N* is the number of solutes (observations) used in the training set, *y*_exp ,*i*_ is the experimental value of the LSER solute parameter (output variable) for solute *i* to be predicted, **x**_***i***_ is the MD vector (input variables) for solute *i*, *b* is the intercept of the linear relationship (the size of *b* is the same as the number of output variables—in this case, since there is only one output variable, it is a scalar), and **w** is the vector of model parameters (whose values are determined by model training). The regularization term  in eq 3 penalizes models with too many nonzero or large elements in **w**. This avoids giving great importance to MDs that are not relevant for the prediction of the output variable (as likely when using ordinary least-squares estimators) and mitigates overfitting through the regularization hyperparameter α.^66^ In this work, the value of α was set via 10-fold cross-validation (CV), i.e., by randomly dividing the training data set into 10-fold and evaluating model performance on each fold at a time, using the remaining 9-fold for calibration. The training data set was obtained dividing the initial data set comprising 6401 molecules using an 80:20 split. Hence, 5120 molecules were used for QSPR model training, while the remaining 1281 were used for testing (i.e., to assess the prediction performance of the QSPR models on unseen molecules; see section: QSPR Prediction Performance and Removal of Redundant MDs). Note that ridge regression can set many elements in **w** to low values (indicating their limited relevance) but does not generally set them to zero (unlike lasso regression). Hence, the number of input parameters was not directly reduced through ridge regression, but was instead addressed as described below.

### QSPR Prediction Performance and Removal of Redundant MDs

**Figure 2:**
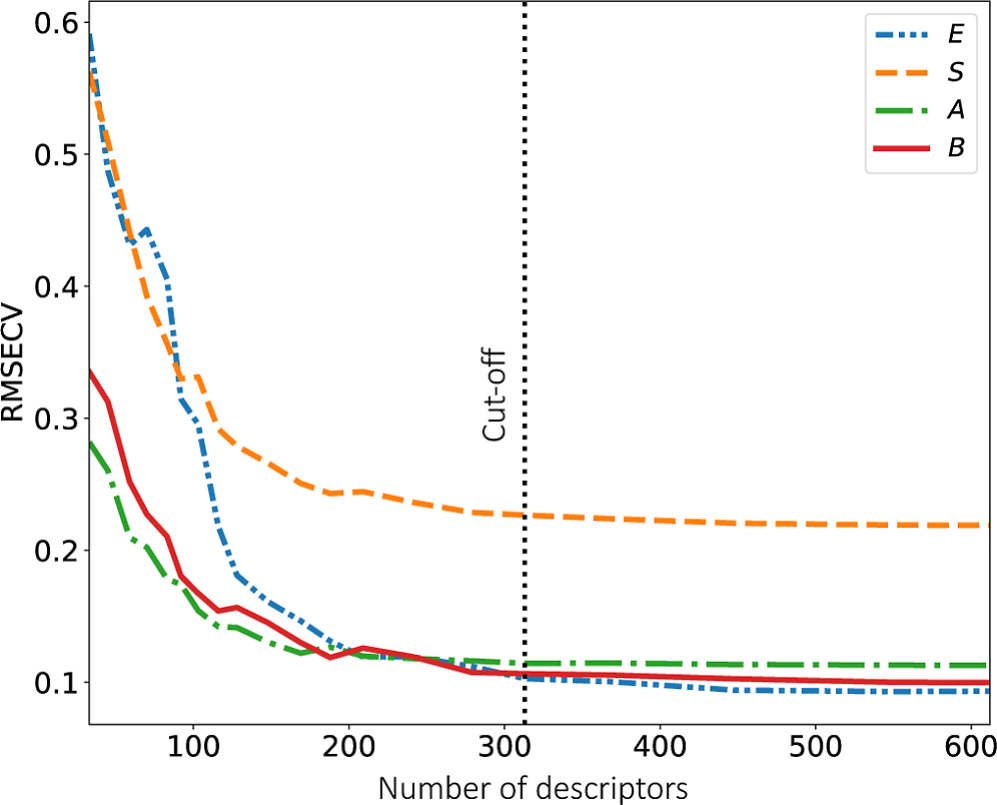
RMSECV decrease with the number of MDs inputs to the QSPR models, for the solute parameters *E* (dash-dot-dotted blue line), *S* (dashed orange line), *A* (dash-dotted green line), and *B* (solid red line). The dotted black line marks the chosen cutoff at 313 MDs.

To reduce the number of QSPR model inputs (i.e., the solute MDs) and thereby reduce the number of model parameters to increase robustness, the number of MDs used was further reduced via a pairwise correlation method as detailed in the Supporting Information. The basic concept is that no significant information is lost when one of the two highly correlated MDs is removed. Therefore, the pairwise correlation coefficients between all (initially, 612) MDs were calculated. For each pair of MDs with a higher correlation coefficient than a set threshold, that with the highest correlation coefficient with the other remaining MDs was removed. Thus, the pairwise correlation threshold was increased stepwise to successively include more MDs. In each step, the predictive performance of the QSPR models was quantified through CV (which was carried out to optimize α). Figure 2 illustrates the impact of MD reduction on QSPR predictive performance, showing that the predictive accuracy remains largely consistent even after removing half of the initial MDs. Based on this analysis, we selected 313 MDs (corresponding to a correlation threshold of 0.85) as inputs for the QSPR models. This selection balances predictive power with a significantly simplified and more robust model.

**Figure 3:**
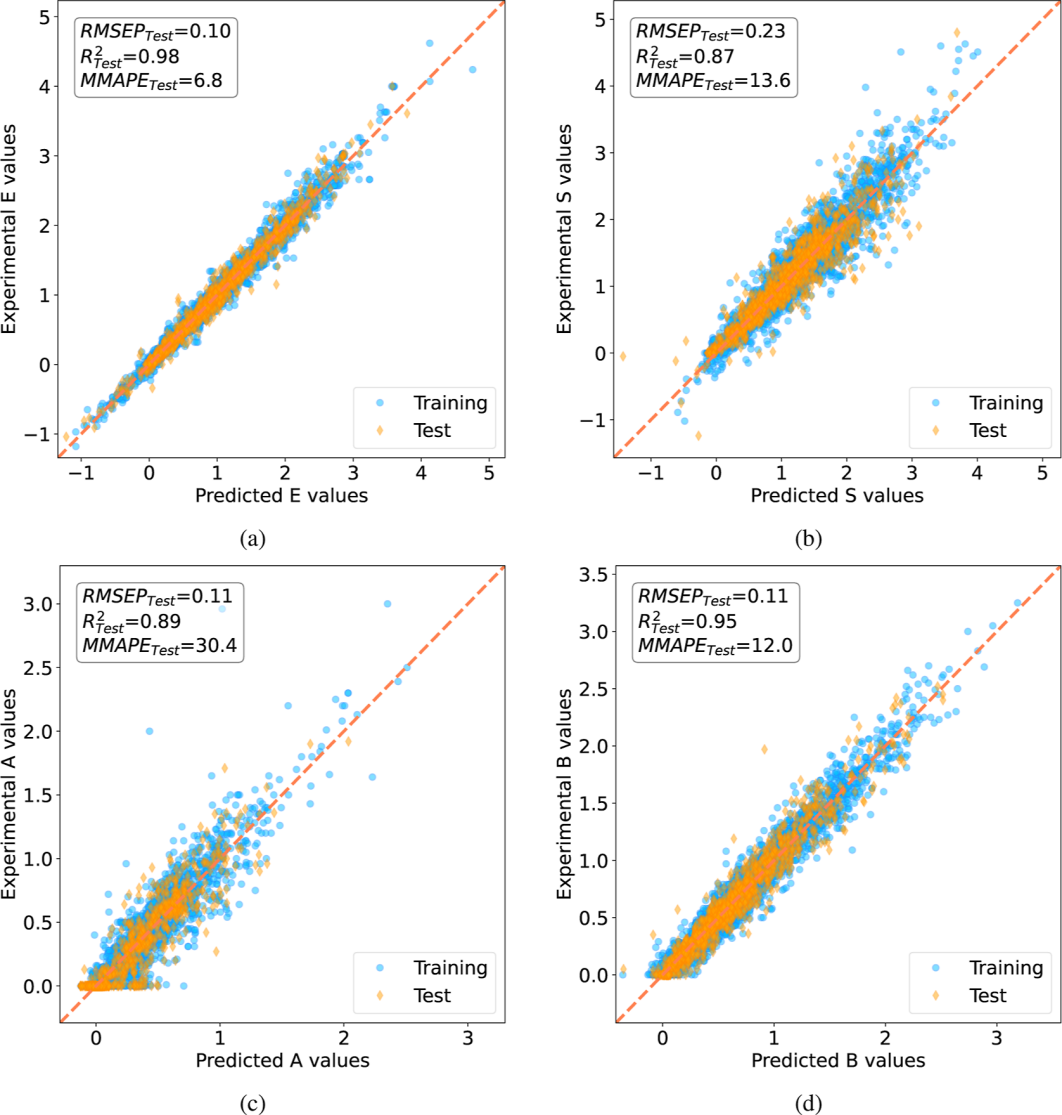
Parity plots comparing experimental and predicted solute parameters (a) *E*, (b) *S*, (c) *A*, and (d) *B*; training (turquoise dots), test (orange diamonds).

Figure 3 shows the parity plots (= predicted vs actual values, with a perfect model aligning points along the **y = x** line) comparing the experimental and QSPR-predicted LSER solute parameters for all training and test solutes. The parity plots include the root mean squared error of prediction (RMSEP, see eq 6), the coefficient of determination *R*^2^ (see eq 7), and a modified mean absolute percentage error (MMAPE, see eq 8), evaluated on the test data set. The criteria and equations used as error indicators, hence to evaluate the predictive capabilities of the QSPR models, are explained in section: Summary of Performance Error Indicators Used to Evaluate Predictive Capabilities. Figure 3 suggests the absence of overfitting. Notably, the Topliss–Costello rule was followed, which recommends a minimum ratio of 5:1 between training observations and input variables to prevent model overfitting.^68,69^ While the general applicability of this rule is debatable, the QSPR models developed here, with a ratio of 16:1 (5120 training molecules and 313 input MDs), comfortably exceed this threshold.

### LSS Theory Parameters Predicted via LSERs

The prediction of the two LSS theory parameters, *k*_w_ and *S*_S,_ by using LSERs was recently demonstrated by Poole and Atapattu.^70^ Their work relied on experimental retention factors of small molecule solutes in different chromatographic systems, including 17 different columns (i.e., stationary phases) for water–methanol mixtures as mobile phase and 15 columns for water–acetonitrile mixtures as mobile phase. In particular, instead of relying on LSERs for retention time/factor prediction (recall eq 2), Poole and Atapattu^70^ used the following two LSERs
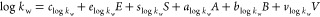
4
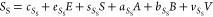
5

This LSER formulation involves two equations and 12 system parameters, namely, , , , , , , and *c*_*S*_S__, *e*_*S*_S__, *s*_*S*_S__, *a*_*S*_S__, *b*_*S*_S__, and *v*_*S*_S__. The advantage of this combination of LSER and LSS theory is that, while the system parameters featuring in eq 2 are functions of the volume fraction of organic modifier (that is, they depend on ϕ and need recalibration if ϕ changes), the system parameters in eqs 4 and 5 are not. They depend on the stationary phase and on the constituents of the mobile phase but not on ϕ. These 6 × 2 = 12 LSER system parameters were obtained via least-squares regression using the values of log  *k*_w_ and *S*_S_ found by applying the LSS theory, i.e., by fitting eq 1 to experimental retention factors (assumed as “true values”, i.e., with no experimental uncertainty associated; see Development of LSERs).

In this work, the LSER system parameters (in eqs 4 and 5) were not taken directly from Poole and Atapattu,^70^ but were obtained likewise via least-squares regression using a reduced (by one) data set for training (see section: Retention Data for LSER Model Development). Specifically, to guarantee that the solutes tested were not part of the data set used to obtain the LSER system parameters, the latter were fitted each time excluding the solute tested (leave-one-out approach; see section: Proof of Concept Demonstration). It is worth noting that in our work, we considered log  *k*_w_ as the natural logarithm of the retention factor *k*_w_ (however, the same methodology could be applied considering the decimal logarithm, as was done by Poole and Atapattu^70^).

### Retention Data for LSER Model Development

Experimentally obtained retention factors used to calibrate eqs 4 and 5 were previously obtained by Poole and Atapattu for their work,^70,71^ but were not therein available. In particular, retention factor data were kindly provided by Prof. Poole directly (personal reference from Wayne State University, 04 January 2023) for the Kinetex XB-C18 Phenomenex column with a water–acetonitrile mixture as mobile phase. Details of the HPLC setup used to obtain these data were previously published.^71^ The data set we used here is made available for download through this article’s Supporting Information (*PooleAtapattuOriginalData.xlsx*). This data set (hereinafter referred to as the LSER data set) comprises the natural logarithm of retention factors of 48 solutes (see Table 1) at 10, 20, 30, 40, 50, 60, and 70% v/v (water/acetonitrile) fractions with occasionally missing data, for a total of 210 retention factors. Indeed, for some solutes, the retention factors were not available for all seven mobile phase compositions.

**Table 1:** 48 Molecules Included in the LSER Dataset Prior to Selection Based on MAPE Associated with Fitting of the LSS Theory with Experimental Retention Factor data. Solutes Not Selected are Marked With*

benzamide	2-bromoacetophenone
benzophenone*	*N*,*N*-dimethylaniline
benzaldehyde	4-fluoroaniline
2-phenylethanol	diphenylamine
4-chlorophenol	caffeine
vanillin	4-nitroaniline
diethyl phthalate*	p-cresol
benzenesulfonamide	3-nitrophenol
coumarin	quinoline*
methyl salicylate	2-nitrophenol
cinnamyl alcohol*	*p*-xylene
diphenyl ether*	*m*-xylene
4-cyanophenol	iodobenzene
*o*-tolualdehyde	1-phenyl-2-propanol*
3-bromophenol*	indole
2-naphthaldehyde*	*N*,*N*-diethylaniline
naphthalene*	2-methoxybenzaldehyde
8-hydroxyquinoline	phthalimide
toluene	nicotinamide*
2-aminophenol	1,3-dibromobenzene
aniline	4-hydroxybenzaldehyde
2-aminobiphenyl*	pentafluorophenol
anisole	4-nitrophenol
4-aminobenzonitrile	4-hydroxybenzamide*

### Development of LSERs

**Figure 4:**
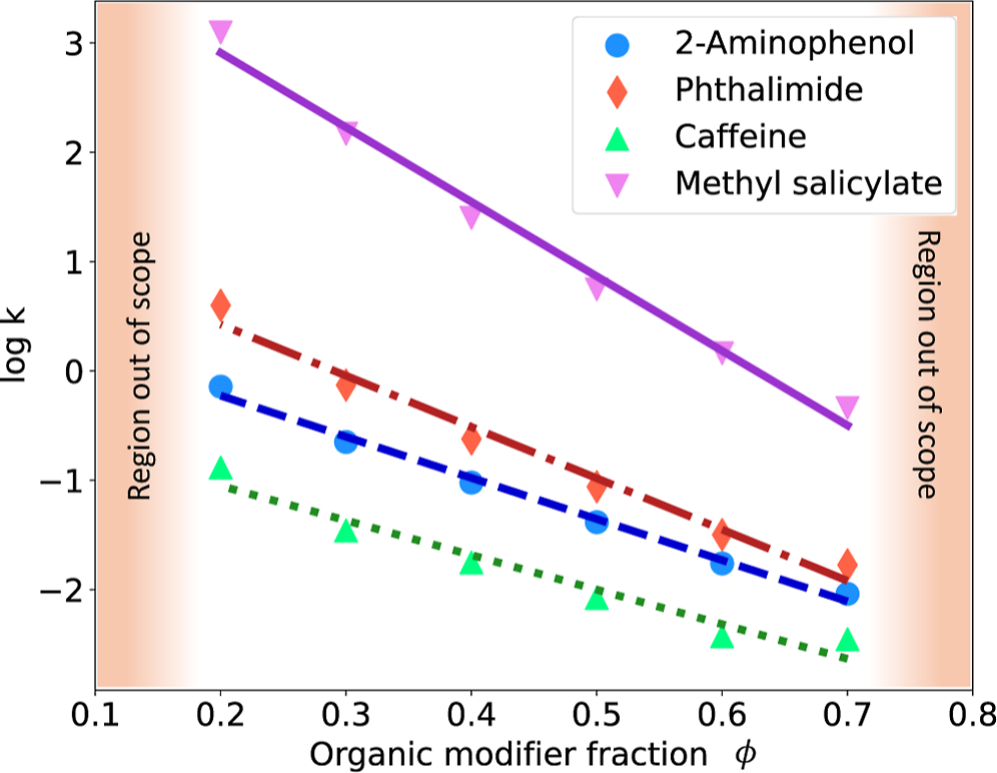
Examples (4 out of the 36) of linear solvent strength theory, i.e., linear fit matching experimental retention factors (symbols as indicated in the legend). The orange region, representing low and high organic modifier fractions, is marked as out of scope due to increased nonlinearity of log  *k* vs ϕ.

The LSS parameters log  *k*_w_ and *S*_S_ (see eq 1) for each solute were obtained via a linear fit of the natural logarithm of experimental retention factors in the LSER data set using ordinary least-squares regression, as the LSS theory linearly relates log  *k* to ϕ. Like Poole and Atapattu,^70^ we restricted the organic modifier volume fraction range to ϕ = 0.2–0.7. Examples of LSS theory fits to experimental retention factors are shown in Figure 4 for four solutes of the LSER data set. For some solutes, the LSS theory failed to adequately represent log  *k* vs ϕ. Hence, not all of the 48 molecules of the LSER data set were used to calibrate the LSERs. To consider only solutes for which the LSS theory is satisfactory, we compared the retention factors at *M* different organic modifier fractions resulting from the linear fit, *k*_fit_ (i.e., *y*_fit_ in eq 9), with the experimental ones, *k*_exp_ (i.e., *y*_exp_ in eq 9), and evaluated for each molecule the mean absolute percentage error (MAPE) (see eq 9) of the fit. We only kept molecules whose MAPE was below 12% (threshold chosen arbitrarily), which reduced the number of solutes from 48 to 36. Table 2 shows the statistics of selected MDs from the 36 molecules kept in the LSER data set. The LSER system-dependent parameters were fitted, as described above, through an ordinary least-squares regression. As already mentioned, the LSER system-dependent parameters in eqs 4 and 5 were determined by minimizing the residual sum of squares between the log  *k*_w_ and *S*_S_ values found by the LSS theory (i.e., the intercept and slope of log  *k* vs ϕ of the solutes used for LSER model calibration) and those calculated by the resulting LSERs.

**Table 2:** Minimum Value, 25th, 50th, and 75th Percentiles, and Maximum Value of Some Selected MDs for the 36 Molecules Left in the LSER Dataset According to Section: Development of LSERs^a^

	MW	nAT	nSK	ARR	%C	%H	%N	%O	%X
min value	92	12	7	0.33	33.3	7.7	0.0	0.0	0.0
25% percentile	116	15	8	0.60	41.6	38.2	0.0	0.0	0.0
50% percentile	132	16	9	0.67	44.4	42.1	1.9	6.3	0.0
75% percentile	150	18	10	0.75	47.8	47.8	6.7	11.8	0.0
max value	236	26	14	1.00	52.9	57.7	16.7	20.0	38.5

a The nomenclature used to indicate the MDs considered is taken from alvaDesc. MW: molecular weight (g/mol); nAT: number of atoms; nSK: number of non-hydrogen atoms; ARR: aromatic ratio within the molecule; %C: % of carbon atoms in the molecule; %H: % of hydrogen atoms in the molecule; %N: % of nitrogen atoms in the molecule; %O: % of oxygen atoms in the molecule; %X: % of halogen atoms in the molecule.

### Summary of Performance Error Indicators Used to Evaluate Predictive Capabilities

Below is a summary of the error indicators used in the above sections. To evaluate model predictive capabilities, we relied on three metrics (or performance indicators): the coefficient of determination *R*^2^, the RMSEP, and a metric analogous to MAPE, which we named modified MAPE (MMAPE). The metrics are defined as follows
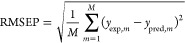
6
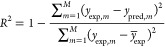
7
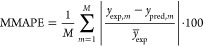
8
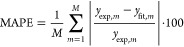
9where *y*_exp ,*m*_ is the *m*-th experimental value of the output variable (in this case, the LSER solute parameter considered), *y*_pred,*m*_ is the *m*-th predicted value of the output variable (i.e., the LSER solute parameter considered), and  is the average value of the experimental output variable (LSER solute parameter) vector, **y**_exp_. As for *M*, it represents the number of test samples. The MMAPE defined by eq 8 was used for QSPR models instead of the MAPE to avoid an inflation of the error for experimental values of the LSER solute parameters that approach (or are equal to) zero.^63^ This is particularly true for parameters *A* and *B*, which were equal to zero for several small molecules used for model training (see Figure 3).

## Methodology at Work

To validate the complete methodology, a leave-one-out approach was used, i.e., one of the 36 solutes kept in the LSER data set was left out at a time, and the LSER system parameters were fitted considering all the remaining molecules. This was repeated 36 times so that all solutes were selected once as a test case. Hence, all 36 solutes (with up to 6 different mobile phase fractions, as ϕ = 0.1 was not considered) were tested using QSPR and LSER models that they did not affect. Recall from section LSER Solute Parameter Prediction via QSPRs, that the 36 solutes left in the LSER data set were not considered for QSPR training and testing, either.

### Proof of Concept Demonstration

The LSERs (eqs 4 and 5) were fitted considering the selected 36 solutes in the LSER data set except one solute at a time (thus, each time 35 molecules were used to calibrate the LSER system parameters; i.e., 2 × 36 LSERs were developed in total). The one solute left out was used to validate the predictive capability of the methodology. For each solute, once its corresponding values of *k*_*w*_ and *S*_*S*_ were determined through two newly developed LSERs, the retention factor *k* was predicted through eq 1 for different ϕ values. These retention factors were then compared with the corresponding experimental values (210 values in total, combining the 36 solutes and the 5 to 6 organic modifier fractions for which data were available).

**Figure 5:**
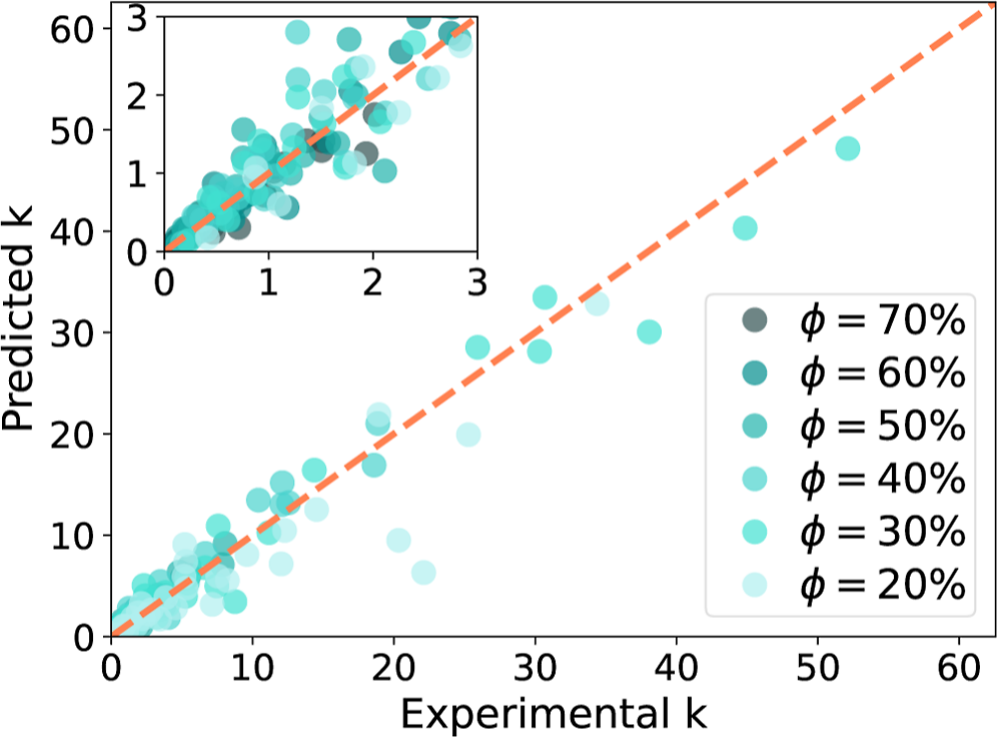
Parity plots comparing the 210 predicted with corresponding experimental retention factors. Each point represents one solute (used for validation, according to a leave-one-out approach) at a given mobile phase composition.

Figure 5 shows the parity plot comparing experimental retention factors with the predicted *k*(ϕ) values. Each data point represents the retention factor of a solute at a given mobile phase composition. These results clearly demonstrate the potential of this methodology. Indeed, even though nothing but SMILES strings was used as input, the retention factors could be estimated reasonably well, with a MAPE below 25% (see Table 3).

**Table 3:** Summary of Error Propagation on the Retention Factor *k*^a^

error	LSS	LSS + LSER	LSS + LSER + QSPR
MAPE	9.1%	17.8%	24.6%
RMSEP	0.98	1.8	1.9
*R*^2^	0.98	0.95	0.94

a MAPE: mean absolute percentage error; RMSEP: root mean squared error of prediction; LSS: linear solvent strength theory; LSER: linear solvation–energy relationships; QSPR: quantitative structure–property relationships.

### Method Error Propagation

**Figure 6:**
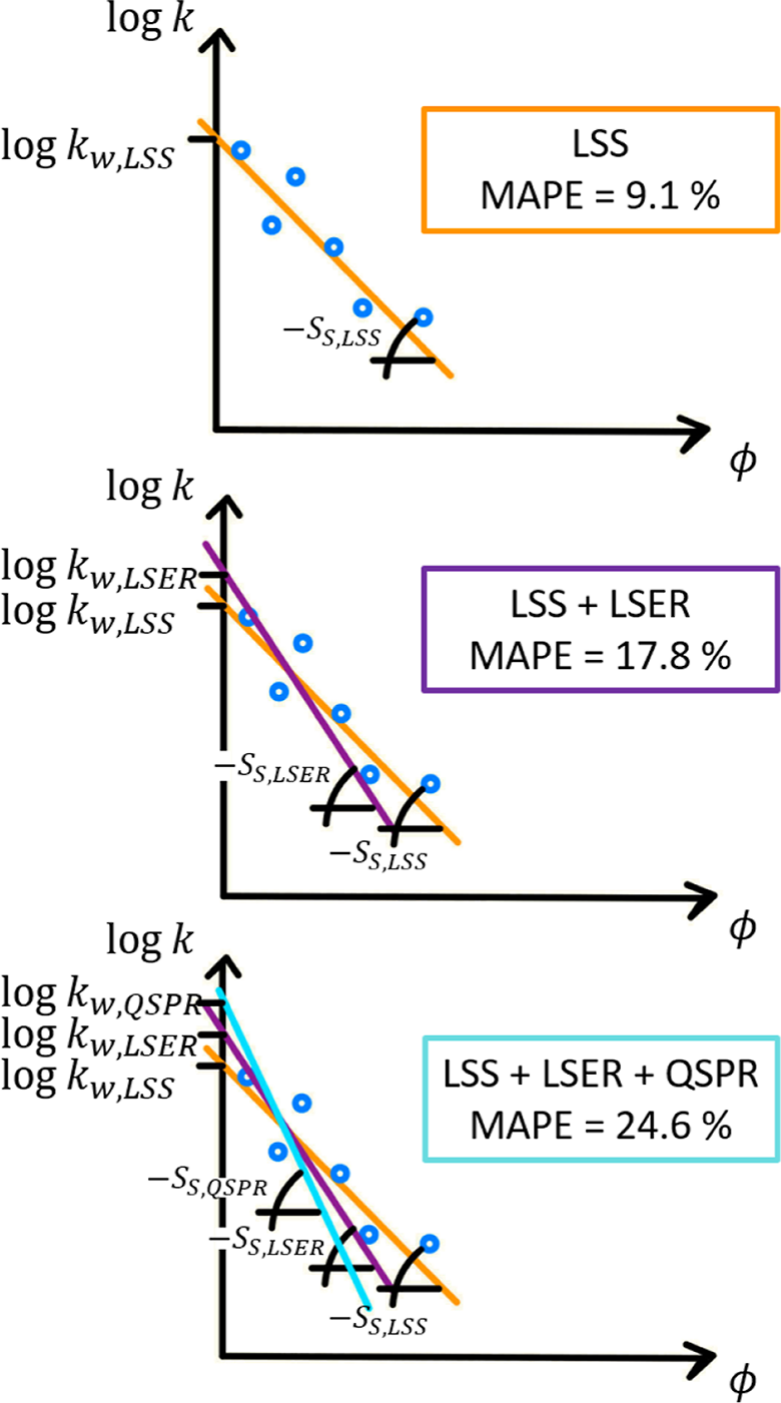
Sketch of error propagation due to the combined use of the (I) linear solvent strength (LSS) theory, (II) linear solvation-energy relationships (LSER), and (III) quantitative structure–property relationships (QSPR). The corresponding parity plots are shown in Figures 5 and S.1.

The predictions achieved are good considering that the methodology aims to predict something as complex as organic modifier-dependent retention factors from the molecular structure of solutes, only. This is even more remarkable considering the limited data available for the LSER calibration. To further improve the accuracy of this data-driven methodology and to assess whether alternative models and larger data sets are required, it is important to understand the origin of the prediction errors made. The retention factor predictions worsen progressively, as expected, when the experimental data are replaced by model predictions. This means that the more experimentally derived information about a solute (e.g., LSER or LSS solute parameters) is provided, the fewer models needed, which leads to more accurate predictions. Figure 6 (top) and Figure S.1 (top, i.e., the corresponding parity plot) show how accurate the predictions are using LSS theory only, i.e., using solely the LSS theory to predict the experimental retention factors of the 36 solutes in the LSER data set. The errors originate from the nonperfect fit using eq 1, which is why errors are larger for higher volume fractions of organic modifier. Figure 6 (middle) and Figure S.1 (bottom, i.e., the corresponding parity plot) show the predictions made using the LSS combined with the LSERs, i.e., LSER predicts the LSS parameters but using experimentally determined LSER solute parameters, instead of using the QSPR for their prediction. Relying on two models instead of one naturally lowers the accuracy and the relative error increase throughout the organic modifier range considered. For the methodology presented here, as already shown in Figure 5, it was still possible to predict the retention factors reasonably well. This was made possible without the luxury of carrying out experiments, but by combining three models, i.e., (a) QSPR, (b) LSER, and (c) LSS theory and using data either publicly available, or provided by the research community. To quantify the contribution of each model (a-c), the considered experimental retention factors were assumed to be the true values (i.e., no experimental uncertainty associated). By using the LSS theory, only, a MAPE (see eq 9) of 9.1% resulted. When determining *k*_*w*_ and *S*_*S*_ via LSER using experimental LSER solute parameters, the MAPE increased to 17.8%. Ultimately, by using all a-c models to facilitate predictions without directly using experimental data, a MAPE of 24.6% was obtained. Table 3 summarizes this error propagation, including also the RMSEP and the coefficient of determination, *R*^2^ (see eqs 6 and 7, respectively) for retention factor predictions. Despite the obvious call for larger data sets to train the LSER models predicting log  *k*_w_ and *S*_S_ (remember that only 36–1 = 35 solutes were used each time for LSER calibration), the methodology error can be assigned to inaccuracies using the LSS theory. This is not unexpected as the LSS theory fails to capture a common nonlinear increase in log  *k* as ϕ → 0. Figure 4 reveals this as the linearity of log  *k* vs ϕ seems valid strictly for 30% < ϕ < 60% only. Hence, to overcome this limitation, the methodology could be extended by introducing more complex (e.g., quadratic) solvent strength models.^21,22^ This, however, would entail additional LSERs to predict the additional parameters of the nonlinear solvent strength model and is therefore not considered here.

## Conclusions and Perspectives

Our methodology offers a practical solution for predicting mobile phase-dependent retention factors using only the molecular structures of solutes. Unlike other data-driven tools that predict solute retention behavior for specific HPLC settings, our approach serves as a promising in-silico tool for optimizing mobile phase profiles. Moreover, since the model inputs consist solely of MDs (obtainable directly from the structural formula of molecules), this methodology can be applied even before any material is synthesized for experimental campaigns. The functionality of our methodology originates from a multimodel data-driven approach that combines (a) quantitative structure–property relationships (QSPR) that use MDs to predict parameters of (b) linear solvation energy relationships (LSER) and (c) classical linear solvent strength (LSS) theory. The methodology’s potential was demonstrated using small molecules whose experimentally determined retention factors were provided by the research community. Although the data set used for this proof of concept was limited in size, the approach demonstrated substantial predictive power. Using only MDs, the methodology predicted mobile phase-dependent retention factors of small molecules in a C-18 stationary phase system with a MAPE of less than 25%. Furthermore, the predictive power of this approach has significant potential for improvement with larger training data sets. Such an increase in available data is anticipated due to the growing number of HPLC databases, advancements in high-throughput HPLC screening capabilities, global digitalization of HPLC method development workflows, and the consolidation of chromatography records across companies. However, it is crucial to emphasize that data set size is not the only factor that matters; the quality of the data set is equally important, particularly the “similarity” between the molecules in the training set and those being predicted. It is clear that any data-driven methodology is only as effective as the data used for training. While we acknowledge that the QSPR models were trained with more than 5000 different small molecules, the data set used for LSER system parameter prediction contained only 35 (i.e., 36–1) solutes with similar size, polarity, and functional group composition. Solutes that differ significantly in these properties are unlikely to allow for accurate prediction of mobile phase dependent elution behavior with the models developed in this work. To make the developed approach truly versatile, it is important to consider that (1) the diversity of molecular features in the data set represents the solutes for which predictions are intended and that (2) this (and all) data-driven approaches should always provide a quantitative measure of similarity between the training data and the target solutes (e.g., molecular features captured through MDs) to indicate the applicability of a model.

When combined with first-principles-based mechanistic transport models, this method can be used to optimize HPLC methods in silico during early development stages. This would significantly reduce the experimental workload and has the potential to render many initial-stage experiments redundant, effectively replacing them with data-driven predictions. Furthermore, by integrating sensible system parameters for LSER to predict LSS parameters for various columns and organic modifiers, our methodology also enables in silico screening of stationary and mobile phases. This capability would further minimize experimental efforts, therefore enhancing efficiency across the HPLC method development workflow.

While the data-driven approach demonstrated remarkable accuracy, it is not yet capable of fully replacing experimental campaigns, especially for complex multicomponent samples prone to coelution. Nevertheless, our results show that this purely data-driven approach provides a reliable initial estimate of elution times across various mobile phase compositions (not considering different pH values) without any prior experimentation required.

In conclusion, the methodology presented in this work provides a promising solution for the prediction of isotherm parameters, offering several advantages. It attains good accuracy without the necessity of any additional experimental data, by utilizing existing databases instead. Furthermore, it may become more powerful with the rapid growth of HPLC databases. The data-driven method presented stands out for its ability to initiate parameter estimation through traditional methods, even in the absence of experimental chromatographic data. This forward-thinking approach gaining its versatility from hybrid models allows for method development before actual samples are obtained, thus enabling a streamlining of the experimental process and potentially saving valuable time and resources.

## Supporting Information Available

Abraham Absolv dataset curation, unsupervised variable reduction in QSPR, and parity plots (PDF)
Data set of retention factors for the Kinetex XB-C18 Phenomenex with a water–acetonitrile mixture as mobile phase used here (XLSX)
