# Supplementary material for: In Silico High-Performance Liquid Chromatography Method Development via Machine Learning
Source: Anal Chem. 2025 Mar 28;97(13):6991–7001. doi: 10.1021/acs.analchem.4c03466 (PMC11983366; doi:10.1021/acs.analchem.4c03466)
Supplement: Supplementary file 1 — ac4c03466_si_001.pdf [file ac4c03466_si_001.pdf]

Supplementary Information for:
In-Silico HPLC Method Development via Machine Learning

Alberto Marchetto[a,b], Monica Tirapelle[a], Luca MAzzei[a], Eva Sorensen[a], Maximilian O. Besenhard[a,*]

[a]*Department of Chemical Engineering, University College London, Torrington Place, London, WC1E 7JE, UK*
[b]*Department of Management, Economics and Industrial Engineering, Politecnico di Milano, Via Rafaele Lambruschini 4/B, Milano, 20156, Italy*
[*]*Corresponding author email address: m.besenhard@ucl.ac.uk*

S.1 Abraham Absolv dataset curation

Solutes with at least one missing value for the four LSER solute parameters were removed from the Abraham Absolv dataset, as well as molecules with missing values for all molecular descriptors and duplicate molecules. Statistics regarding some relevant molecular properties of molecules included in the resulting dataset are shown in Table S.1. As can be seen from Table S.1, the minimum value of the molecular weight found among the remaining molecules was 2.02 g/mol (hydrogen), whereas the maximum was 1,203 g/mol, with the median (50th percentile) at approximately 180 g/mol. Such a range of molecular weights was judged too broad, and the range was therefore narrowed down. In particular, only molecules within the interval 80–400 g/mol were kept in order to include the range of molecular weights of molecules in the Poole dataset (see Table 2). The upper and lower bounds of this range were chosen somewhat arbitrarily, and may not be the best choice. Ultimately, the 36 molecules that were finally kept from the LSER dataset (see Section: Retention data for LSER model development) were removed from the Abraham dataset. As a consequence, 6,401 molecules were left in the Abraham Absolv dataset from the 7,881 initially present. A dataset split was also done to lead to a training set and a test set, following a split ratio of 80:20 (80% of molecules were randomly selected to be part of the calibration set, while the remaining 20% defined the test set). Molecular descriptors that were constant or nearly constant in the training set were discarded, based on a filter for the variance set to 0.01 ($var > 0.01$). Additionally, an MD whose entries were mostly missing was removed following a manual inspection. These steps resulted in a decrease in the number of MDs from 804 to 612. Missing values of MDs were substituted with the median calculated for the same descriptor over the molecules in the training set. Scaling was also applied to normalise the distribution of descriptors in the training set to zero mean and unit variance. The same scaling was then applied to test data.

S.2 Unsupervised variable reduction in QSPRs

In this work, we applied a pairwise correlation method for unsupervised variable reduction in the four QSPR models. We considered threshold values for the pairwise correlation method included in the following list:

[0.1, 0.15, 0.2, 0.25, ..., 0.9, 0.95, 0.99, 1.5]

Table S.1: Minimum value, 25th, 50th and 75th percentiles, and maximum value of some selected molecular descriptors for the molecules included in the Abraham Absolv dataset. The nomenclature used to indicate the molecular descriptors considered is taken from alvaDesc. MW: molecular weight (g/mol); nAT: number of atoms; nSK: number of non-hydrogen atoms; ARR: aromatic ratio within the molecule; C%: % of carbon atoms in the molecule; H%: % of hydrogen atoms in the molecule; N%: % of nitrogen atoms in the molecule; O%: % of oxygen atoms in the molecule; X%: % of halogen atoms in the molecule.

	**MW**	**nAT**	**nSK**	**ARR**	**C%**	**H%**	**N%**	**O%**	**X%**
**Min value**	2	1	0	0.00	0.0	0.0	0.0	0.0	0.0
**25% percentile**	134	18	9	0.00	31.6	40.0	0.0	0.0	0.0
**50% percentile**	178	24	12	0.35	37.8	50.0	0.0	4.8	0.0
**75% percentile**	251	32	16	0.60	43.5	60.0	4.9	9.3	1.7
**Max value**	1203	196	85	1.00	100	100	100	100	100

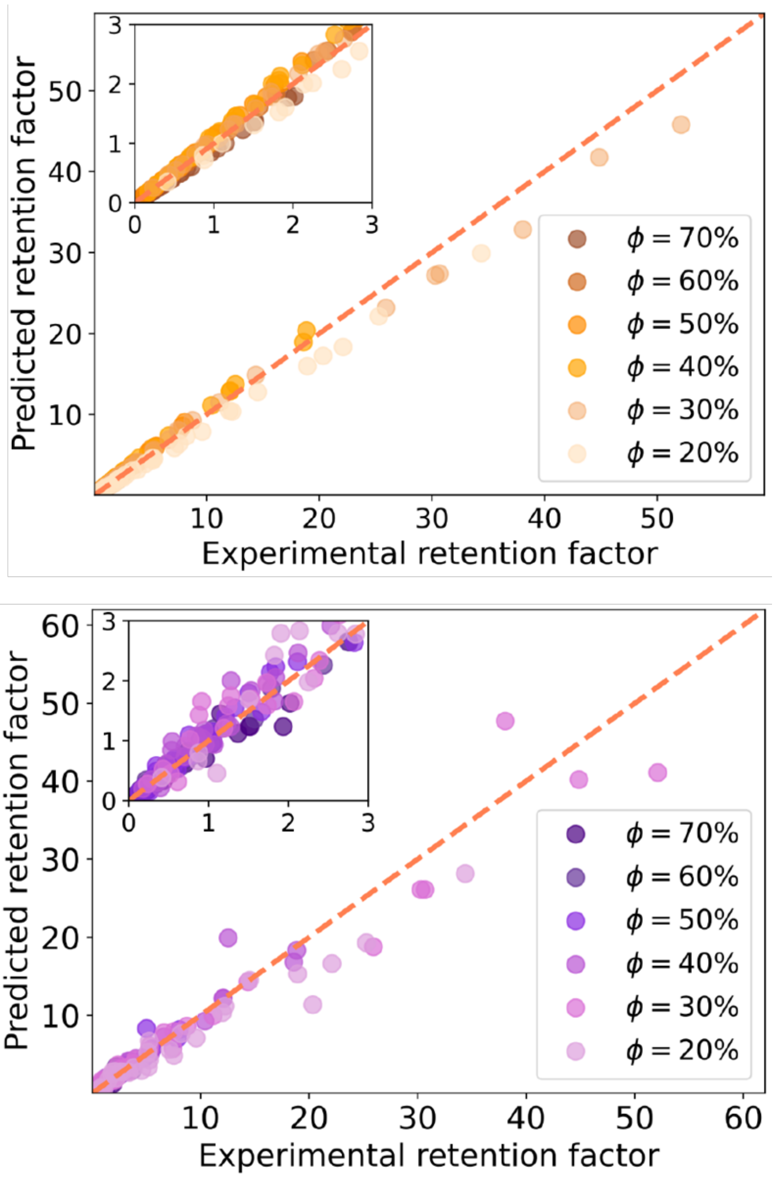

Figure S.1: Parity plots corresponding to Figure 6 (top and middle), i.e., the results of the proof of concept demonstration when using the linear solvent strength (LSS) theory, only (top), and LSS combined with the linear solvation-energy relationships (LSER), with experimental LSER solute parameters (bottom).

in order to consider QSPR predictive performance moving from very few (weakly correlated) molecular descriptors (threshold: 0.1) to all descriptors (threshold: $1.5 > 1$; here, as the threshold is greater than 1, all MDs are kept because correlation coefficients can be at most 1). The pairwise correlation method applied here has some limitations. In particular, a notable one is that variations in the order of the MDs in the MD dataset may determine changes in which MDs are removed.

The effect of MD reduction on QSPR predictive performance (evaluated through CV) are shown in Figure 2, where it can be seen that as more descriptors are included as input variables of the QSPRs, model predictive performance improve. However, for all four parameters E, S, A and B, the improvement in model predictive performance reaches a plateau once approximately 250 to 310 MDs are included by the pairwise correlation method. Once the plateau is reached, including further MDs will not introduce any additional advantage in terms of QSPR predictive capabilities, while making the QSPRs more complex. From Figure 2, we decided to consider a threshold for the pairwise correlation method of 0.85, which corresponds to 313 molecular descriptors as QSPR input variables (the same MDs were selected for each of the four QSPRs).

S.3 Parity plots for LSS and LSS + LSER model use for retention factor prediction

Parity plots in Figure S.1 refer to retention factor predictions by relying on the LSS theory, only (figure above), and by relying on the LSS combined with the LSER, with experimental LSER solute parameters. The parity plot considering also the QSPR model, i.e., all three models of the complete data-driven methodology, is shown in Figure 5.
